# Neutrophils extracellular traps myeloperoxidase and elastase predict cerebral vasospasms after aneurysmal subarachnoid hemorrhage

**DOI:** 10.1016/j.heliyon.2024.e40562

**Published:** 2024-11-20

**Authors:** Saba Sajjad, Michael Hewera, Majeed Rana, Michael Gliem, Igor Fischer, Dilaware Khan

**Affiliations:** a Department of Oral, Maxillofacial and Facial Plastic Surgery, University Hospital Düsseldorf, Düsseldorf, Germany; b Department of Neurosurgery, Medical Faculty and University Hospital Düsseldorf, Heinrich-Heine-University, Düsseldorf, Germany; c Department of Neurology, University Hospital Düsseldorf, Düsseldorf, Germany

**Keywords:** aSAH, Cerebral vasospasm, Myeloperoxidase, Elastase, Predictive model

## Abstract

Aneurysmal subarachnoid hemorrhage (aSAH) is a highly fatal and morbid disease. Despite successful coiling or clipping of a ruptured aneurysm, the patients suffer post-aSAH complications, including early brain injury, cerebral vasospasm (CVS), delayed cerebral ischemia (DCI), and systemic infections that mainly determine the clinical outcomes. Diagnostic biomarkers to predict accurately post-aSAH complications are needed. In this prospective exploratory study, we investigated the predictive value of neutrophil extracellular traps (NETs) components for CVS after aSAH. In the study, 62 patients with aSAH, 17 patients with unruptured cerebral aneurysms, and 12 healthy controls were included. The serum levels of myeloperoxidase (MPO), elastase (ELA), and citrullinated histone H3 (cH3) on day 1 and day 4 of hospital admission were measured with ELISA. Data were scaled using the Yeo-Johnson transformation. Values in two groups were compared using a *t*-test and in multiple groups using ANOVA. Logistic regression was used to model the outcome probability, including CVS, as the function of ELISA values. Among the patients with aneurysms, those who suffered aSAH had significantly higher levels of MPO (113.9 ± 294.4 vs. 422.3 ± 319.0 ng/ml, p < 0.05), ELA (84.8 ± 221.0 vs. 199.2 ± 218.9 ng/ml, p < 0.05), and cH3 (0.0 ± 0.0 vs. 2.8 ± 1.5, ng/ml, p < 0.05) on day one after aSAH, suggesting the involvement of NETs components in pathophysiology of aSAH and the events following aSAH. Individually, MPO and ELA levels taken on day 1 after SAH did not differ between patients with CVS and patients without CVS. However, when taken together into a logistic model, they allowed for predicting CVS with high sensitivity (91 %) and specificity (79 %). MPO and ELA, along with other clinical parameters, can be used as early predictors of CVS in aSAH patients and can serve as guidance during treatment decisions in the management of aSAH.

## 1 Introduction

Aneurysmal subarachnoid hemorrhage (aSAH) is a highly fatal and morbid disease, killing around 40 % of the patients [1,2]. The majority of the survivors suffer diverse neurological disabilities, posing a very high socioeconomic burden at the community level [3]. Despite successful coiling or clipping of a ruptured aneurysm, the early brain injury (EBI) and post-SAH complications in the delayed phase, including cerebral vasospasm (CVS), delayed cerebral ischemia (DCI), hydrocephalus, epilepsy, and systemic infections mainly determine the clinical outcomes [4].

There is strong evidence that cellular and molecular mediators of inflammation contribute to cerebral aneurysm formation, cerebral aneurysm rupture, and post-aSAH complications, prognosis, and clinical outcomes [[5], [6], [7], [8], [9]]. The exact underlying mechanisms are not fully understood. However, it is well-known that pro-inflammatory response plays a crucial role in initiating EBI, CVS, and DCI following aSAH [5]. The infiltration of inflammatory cells following SAH contributes heavily to pro-inflammatory response. Previous studies have shown the infiltration of innate immune cells; neutrophils, and monocytes/macrophages into the brain after SAH [5,10]. Provencio et al. showed that myeloid cell depletion using myeloid cell-depleting monoclonal antibodies against Ly6G/C reduced angiographic vasospasm and improved behavioral scores in experimental animal studies [11]. Neutrophils are the first responders to infection and defend the body against microbial invasion. The count of neutrophils in the peripheral system and the high neutrophil to lymphocyte ratio have been associated with poor prognosis, complications, and mortality in neurovascular diseases such as aSAH and ischemic stroke [[12], [13], [14], [15], [16]]. A high number of neutrophils has been observed in the brain microvessels as early as 10 min following SAH [10]. The neutrophil infiltration was higher in the ruptured aneurysm compared to the unruptured aneurysm [17]. Interestingly, these infiltrated neutrophils were found close to the site of rupture and rarely observed in other parts of the aneurysmal wall [28], suggesting their crucial role in aneurysm rupture. The level of neutrophil enzymes in aneurysm walls has been linked to a higher risk of aneurysm rupture [18]. The neutrophil proteases are needed for anti-microbial functions, however, these enzymes can elicit pro-inflammatory responses [19] and can cause tissue damage and tissue remodeling, which are known to play an important role in aneurysm formation and rupture [9].

The infiltration of neutrophils results in early brain injury following SAH. Neutrophils can gradually infiltrate and accumulate in the local brain parenchyma, exacerbating local brain tissue damage. Neutrophil removal, depletion, or inactivation ameliorated hypoperfusion, attenuated DCI, neuronal loss, production of pro-inflammatory cytokines, and generation of ROS [5]. Neutrophils contribute to inflammation by releasing pro-inflammatory cytokines and discharging extracellular traps (NETs). NETs comprise DNA, citrullinated histone 3 (cH3), and neutrophil granule proteins such as myeloperoxidase (MPO) and elastase. Elevated NETs formation was observed in the subarachnoid space on the ipsilateral side on day one in the experimental SAH mouse model [20]. NETs can impair the blood-brain barrier [21,22], consequently exposing brain tissue to circulating immune cells and neurotoxic blood components such as hemoglobin, thrombin, free iron, fibrinogen, methemoglobin, and hemin, resulting in enhanced inflammation, oxidative stress, and brain tissue damage [23]. NETs can also cause brain damage by triggering neuronal and cellular degeneration in the surrounding tissue [[24], [25], [26]]. Additionally, NETs can increase ROS generation [27], which exacerbates inflammation and strengthens NETs formation [28]. Moreover, the inhibition of NETs formation dampened neuroinflammation, attenuated neuronal apoptosis, mitigated DCI, and prevented early brain injury in mice after SAH and traumatic brain injury [[24], [25], [26],[29], [30], [31]].

In this exploratory study, we focused on three major components of NETs: elastase, myeloperoxidase, and citrullinated histone H3, and investigated whether these NETs components can predict cerebral vasospasms following aSAH.

## 2 Materials and methods

### 2.1 Study variables

World Federation of Neurosurgical Societies (WFNS)

The WFNS is used to grade the clinical severity of SAH based on the presence of focal neurological deficits and the Glasgow Coma Scale (GCS). It ranges from 1, meaning no focal deficit, to 5, meaning deep coma.

### 2.2 Modified Rankin Scale (mRS)

The mRS is used to measure the outcome in patients after aSAH. The 7-point mRS scale ranges from 0 to 6, where mRS 0 means no symptoms, worsening to mRS 6. For example, mRS 1 means symptoms with no significant disability, mRS 2 means a slight disability, mRS 5 means severe disability, and mRS 6 means death.

CVS.

CVS is the reversible narrowing of cerebral arteries, which results in reduced blood flow and possible stroke in the corresponding brain region.

### 2.3 Study type and population

During 2020–2022, we prospectively enrolled 62 aSAH patients and 17 patients with aneurysms. Twelve healthy volunteer co-workers were enrolled in the study. Informed consent was obtained from all patients and healthy controls. In cases where patients could not give informed consent, the informed consent was obtained from their authorized proxy.

## 3 Data collection

The summary of the patient demographics is given in Table 1.

**Table 1:** Cohort demographics, total and by outcome group.

Levels	Total	mRS≤2	3≤mRS≤5	mRS = 6	p-Value
sex	n (%)	n (%)	n (%)	n (%)	p
0	39 (35 %)	12 (32 %)	10 (33 %)	3 (27 %)	0.900
1	71 (65 %)	25 (68 %)	20 (67 %)	8 (73 %)	
WFNS	n (%)	n (%)	n (%)	n (%)	p
1	23 (29 %)	19 (51 %)	4 (13 %)	0 (0 %)	1,00E-01
2	11 (14 %)	8 (22 %)	2 (7 %)	1 (9 %)	
3	5 (6 %)	2 (5 %)	3 (10 %)	0 (0 %)	
4	19 (24 %)	6 (16 %)	9 (30 %)	3 (27 %)	
5	21 (27 %)	2 (5 %)	12 (40 %)	7 (64 %)	
vasospasm_DSA_1	n (%)	n (%)	n (%)	n (%)	p
0	64 (82 %)	30 (81 %)	25 (83 %)	9 (82 %)	0.900
1	13 (17 %)	6 (16 %)	5 (17 %)	2 (18 %)	
2	1 (1 %)	1 (3 %)	0 (0 %)	0 (0 %)	
VPS	n (%)	n (%)	n (%)	n (%)	p
0	50 (65 %)	30 (83 %)	9 (30 %)	11 (100 %)	1,00E-03
1	27 (35 %)	6 (17 %)	21 (70 %)	0 (0 %)	
DTT	n (%)	n (%)	n (%)	n (%)	p
0	48 (62 %)	37 (100 %)	6 (21 %)	5 (45 %)	2,00E-07
1	29 (38 %)	0 (0 %)	23 (79 %)	6 (55 %)	
infarction	n (%)	n (%)	n (%)	n (%)	p
0	45 (58 %)	27 (73 %)	14 (47 %)	4 (36 %)	0.030
1	33 (42 %)	10 (27 %)	16 (53 %)	7 (64 %)	
EVD	n (%)	n (%)	n (%)	n (%)	p
0	21 (27 %)	20 (54 %)	1 (3 %)	0 (0 %)	2,00E-03
1	58 (73 %)	17 (46 %)	29 (97 %)	11 (100 %)	

### 3.1 Serum collection and analysis

#### 3.1.1 Serum extraction

The blood samples were collected from patients within 24 h of admission and on the fourth day after admission. Serum was extracted, by centrifuging patients’ blood at 2000 rcf for 10 min. The serum was stored at −80 °C directly.

## 4 ELISA

For Performing the ELISAS, the following Kits were used: Myeloperoxidase (Biotechne, DY3174), Neutrophil Elastase (Biotechne, DY9167-05), Citrullinated Histone H3 (Cayman Chemicals, Cay501620-96). For all Kits the DuoSet ELISA Ancillary Reagent Kit 2 was used (Biotechne, DY008B). Before Performing the ELISAs, one serum Sample was diluted in a geometric dilution with Reagent Dilution Buffer, to find the suitable dilution for each ELISA kit. Citrullinated Histone H3 ELISA was performed according to the manual with 1:10 diluted serum. Neutrophil Elastase and Myeloperoxidase ELISAs were performed according to their manuals with 1:128 diluted serum. All Dilutions were done with a Reagent Dilution Buffer.

### 4.1 Data analysis

The levels of cH3, ELA, and MPO were visually inspected and checked for normality using the Shapiro-Wilk test. As they showed to be highly skewed, they were scaled using the Yeo-Johnson power transformation [32], which is an improvement over the older and perhaps better-known Box-Cox transformation. A T-test on the transformed values was used to check for their association with dichotomous variables: sex, age (<60/≥ 60), WFNS (≤3/≥ 4), the presence of CVS, and infarction. One-way ANOVA was used for testing the association with the trichotomized outcome (mRS ≤2, 3 ≤ mRS ≤5, mRS = 6), separately for samples collected on day one and day four after SAH. If ANOVA showed significant differences between outcome groups, post-hoc *t*-test was used to identify them. A summary of the stratified values is given in Table 2, Table 3, Table 4, Table 5.

**Table 2:** ELISA values stratified by age.

Variable	Total	age<60	Age≥60	p-Value
	mean (sd)	mean (sd)	mean (sd)	
Myeloperoxidase (day 1)	349.2 (335.3)	424.8 (330.4)	426.1 (310.0)	0.700
Myeloperoxidase (day 4)	6.9 (8.6)	7.5 (8.6)	6.8 (9.0)	1.000
Elastase (day 1)	177.4 (224.4)	200.6 (228.9)	203.9 (207.4)	0.800
Elastase (day 4)	3.1 (2.2)	3.3 (2.3)	3.1 (2.1)	0.700
Citrullinated Histone 3 (day 1)	2.3 (1.8)	2.7 (1.5)	2.8 (1.7)	1.000
Citrullinated Histone 3 (day 4)	57.7 (68.8)	59.0 (76.1)	61.5 (60.3)	0.900
Platelet Factor 4 (day 1)	6.9 (2.4)	7.5 (2.5)	6.7 (1.9)	0.300
Stromal cell-Derived Factor (day 1)	80.0 (202.9)	94.3 (273.3)	44.2 (56.5)	0.400

**Table 3:** ELISA values stratified by group.

Variable	Total	Aneurysm	Healthy	SAH	p-Value
	mean (sd)	mean (sd)	mean (sd)	mean (sd)	
Myeloperoxidase (day 1)	349.2 (335.3)	113.9 (294.4)	298.4 (334.2)	422.3 (319.0)	5,00E-16
Myeloperoxidase (day 4)	6.9 (8.6)	nan (nan)	nan (nan)	6.9 (8.6)	nan
Elastase (day 1)	177.4 (224.4)	84.8 (221.0)	193.8 (243.1)	199.2 (218.9)	7,00E-14
Elastase (day 4)	3.1 (2.2)	nan (nan)	nan (nan)	3.1 (2.2)	nan
Citrullinated Histone 3 (day 1)	2.3 (1.8)	0.0 (0.0)	4.8 (2.2)	2.8 (1.5)	5,00E-16
Citrulinated Histone 3 (day 4)	57.7 (68.8)	nan (nan)	nan (nan)	57.7 (68.8)	nan
Platelet Factor 4 (day 1)	6.9 (2.4)	5.3 (0.8)	6.0 (2.4)	7.2 (2.3)	0.070
Stromal cell-Derived Factor (day 1)	80.0 (202.9)	84.4 (21.9)	103.2 (128.2)	75.3 (220.8)	0.070

**Table 4:** ELISA values stratified by initial status.

Variable	Total	WFNS≤3	WFNS≥3	p-Value
	mean (sd)	mean (sd)	mean (sd)	
Myeloperoxidase (day 1)	422.3 (319.0)	368.0 (227.7)	475.5 (381.0)	0.200
Myeloperoxidase (day 4)	6.9 (8.6)	4.7 (6.2)	9.4 (9.9)	0.060
Elastase (day 1)	199.2 (218.9)	132.9 (110.4)	262.3 (270.8)	0.060
Elastase (day 4)	3.1 (2.2)	2.5 (1.8)	3.8 (2.4)	0.030
Citrullinated Histone 3 (day 1)	2.8 (1.5)	2.8 (1.6)	2.7 (1.5)	0.700
Citrullinated Histone 3 (day 4)	57.7 (68.8)	69.1 (89.0)	52.0 (47.9)	0.600
Platelet Factor 4 (day 1)	7.2 (2.3)	7.3 (2.1)	7.2 (2.6)	0.600
Stromal cell-Derived Factor (day 1)	75.3 (220.8)	27.5 (36.8)	119.7 (298.3)	0.002

**Table 5:** ELISA values stratified outcome.

Variable	Total	mRS≤2	3≤mRS≤5	mRS = 6	p-Value
	mean (sd)	mean (sd)	mean (sd)	mean (sd)	
Myeloperoxidase (day 1)	422.3 (319.0)	396.5 (323.1)	475.1 (310.0)	363.7 (365.7)	0.300
Myeloperoxidase (day 4)	6.9 (8.6)	4.9 (6.7)	9.0 (9.0)	10.7 (12.8)	0.100
Elastase (day 1)	199.2 (218.9)	159.7 (218.3)	259.5 (234.0)	161.6 (141.9)	0.200
Elastase (day 4)	3.1 (2.2)	2.7 (2.0)	3.5 (2.1)	3.9 (3.3)	0.300
Citrullinated Histone 3 (day 1)	2.8 (1.5)	3.0 (1.8)	2.6 (1.3)	2.2 (0.8)	0.800
Citrullinated Histone 3 (day 4)	57.7 (68.8)	69.1 (88.8)	47.3 (37.5)	75.5 (74.4)	0.700
Platelet Factor 4 (day 1)	7.2 (2.3)	7.3 (2.1)	7.1 (2.6)	7.1 (2.4)	0.800
Stromal cell-Derived Factor (day 1)	75.3 (220.8)	32.4 (35.7)	73.6 (71.6)	240.6 (607.5)	0.200

Simple and multiple logistic regression was used to model the probability of SAH among aneurysm patients and the probability of CVS and infarction among SAH patients, depending on cH3, MPO, and ELA. Model performance was measured using McFadden's pseudo-R^2^ and, for discrete predictions, using sensitivity, specificity, accuracy, F_1_-score, and the area under the sensitivity-specificity curve.

All computations were performed in Python (www.python.org), using NumPy [33], SciPy [34], scikit-learn [35] and Statsmodels [36] libraries.

## 5 Results

**Fig. 1:**
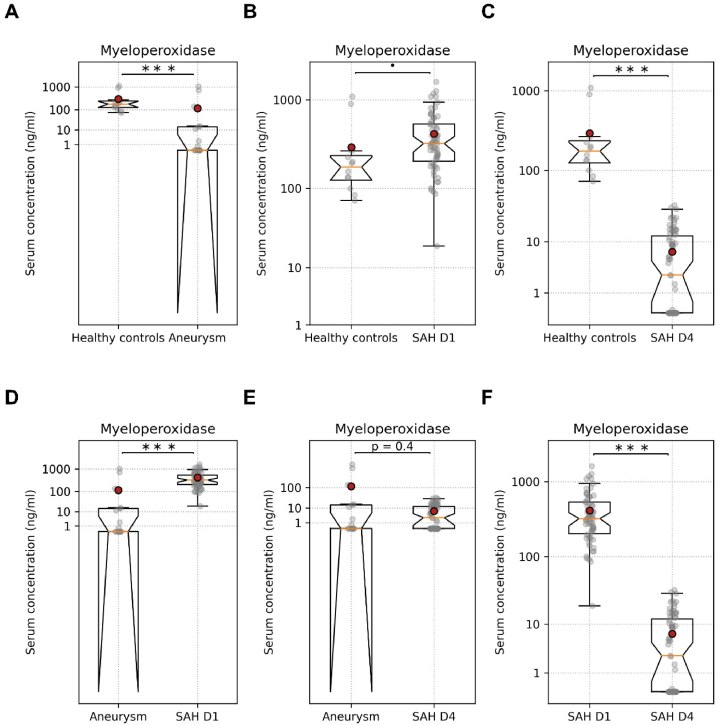
Serum levels of MPO. (A) Serum MPO levels in healthy controls and aneurysm patients. (B) Serum MPO levels in healthy controls and aSAH patients on day 1. (C) Serum MPO levels in healthy controls and aSAH patients on day 4. (D) Serum MPO levels in aneurysm controls and aSAH patients on day 1. (E) Serum MPO levels in aneurysm controls and aSAH patients on day 4. (F) Serum MPO levels in aSAH patients on day 1 and aSAH patients on day 4. Unpaired *t*-test for all data and paired *t*-test for aSAH day 1 and aSAH day 4. •p < 0.1, ∗p < 0.05, ∗∗∗∗p < 0.0001. SAH D1 = post aneurysmal subarachnoid hemorrhage day 1, SAH D4 = post aneurysmal subarachnoid hemorrhage day 4.

No significant differences in serum MPO, ELA, and cH3 levels were detected for different sexes and age groups (<60/≥ 60; Table 1, Table 2). The serum MPO levels were significantly lower in patients with aneurysm control than in healthy controls and in aSAH patients on day 1 (Fig. 1A–D; Table 3). The serum MPO levels in aSAH patients on day 1 showed a tendency to increase as compared to healthy controls, but the difference was not significant (Fig. 1B; Table 3). The serum MPO levels were significantly lower in aSAH patients on day 4 as compared to healthy control and aSAH patients on day 1 (Fig. 1C–F; Table 3). There was no difference in serum MPO levels in patients with aneurysm as compared to aSAH patients on day 4 (Fig. 1E; Table 3).

**Fig. 2:**
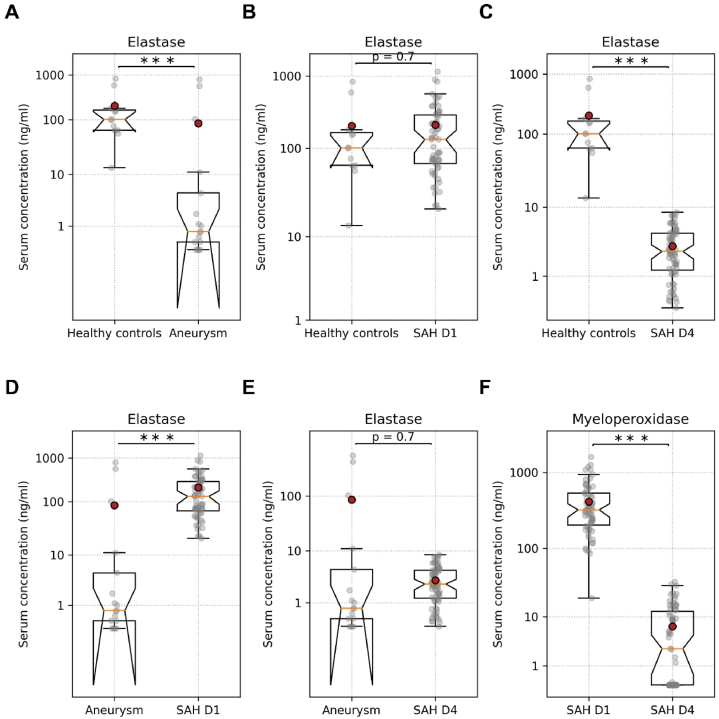
Serum levels of elastase. (A) Serum elastase levels in healthy controls and aneurysm patients. (B) Serum elastase levels in healthy controls and aSAH patients on day 1. (C) Serum elastase levels in healthy controls and aSAH patients on day 4. (D) Serum elastase levels in aneurysm controls and aSAH patients on day 1. (E) Serum elastase levels in aneurysm controls and aSAH patients on day 4. (F) Serum elastase levels in aSAH patients on day 1 and aSAH patients on day 4. Unpaired *t*-test for all data and paired *t*-test for aSAH day 1 and aSAH day 4. ∗∗∗p < 0.001. SAH day 1 = post aneurysmal subarachnoid hemorrhage day 1, SAH D4 = post aneurysmal subarachnoid hemorrhage day 4.

The serum elastase levels were significantly lower in aneurysm patients than in healthy controls and aSAH patients on day 1 (Fig. 2A–D; Table 3). There was no difference in serum elastase levels between healthy control and aSAH patients on day 1 (Fig. 2B; Table 3). The aSAH patients on day 4 had significantly lower serum elastase levels compared to healthy controls and aSAH patients on day 1 (Fig. 2C–F; Table 3). The serum elastase levels did not differ between aneurysm patients and aSAH patients on day 4 (Fig. 2E; Table 3).

**Fig. 3:**
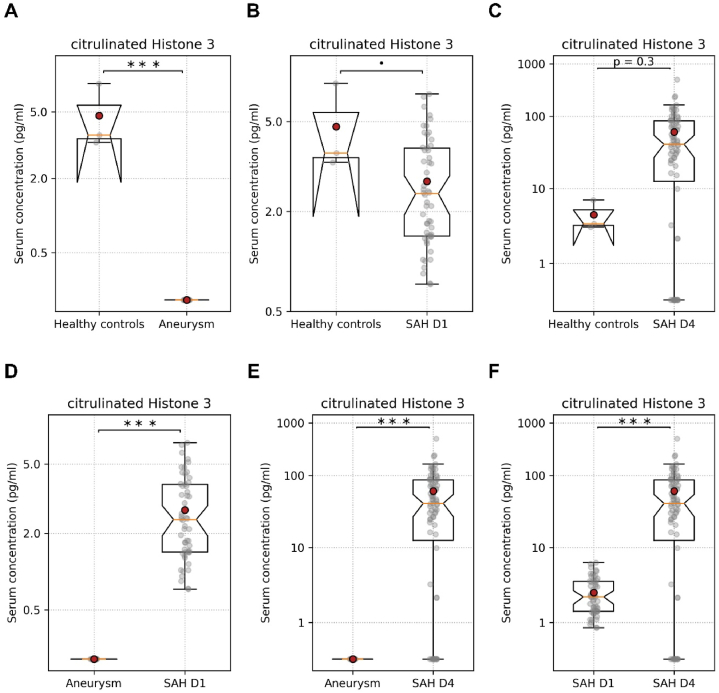
Serum levels of cH3. (A) Serum cH3 levels in healthy controls and aneurysm patients. (B) Serum cH3 levels in healthy controls and aSAH patients on day 1. (C) Serum cH3 levels in healthy controls and aSAH patients on day 4. (D) Serum cH3 levels in aneurysm controls and aSAH patients on day 1. (E) Serum cH3 levels in aneurysm controls and aSAH patients on day 4. (F) Serum cH3 levels in aSAH patients on day 1 and aSAH patients on day 4. Unpaired *t*-test for all data and paired *t*-test for SAH day 1 and SAH day 4. •p < 0.1, ∗∗∗p < 0.001. SAH D1 = post aneurysmal subarachnoid hemorrhage day 1, SAH D4 = post aneurysmal subarachnoid hemorrhage day 4.

The serum cH3 levels were significantly lower in aneurysm patients than healthy controls, aSAH patients on day 1, and aSAH patients on day 4 (Fig. 3A–D, E; Table 3). The serum cH3 levels showed a tendency to decrease in aSAH patients on day 1 after aSAH when compared to healthy controls, but the difference was not significant (Fig. 3B; Table 3). There was no difference in serum cH3 levels between healthy controls and aSAH patients on day 4 (Fig. 3C; Table 3). The serum cH3 levels were significantly higher in aSAH patients on day 4 than in patients with aneurysm and aSAH patients on day 1 (Fig. 3E and F; Table 1).

**Fig. 4:**
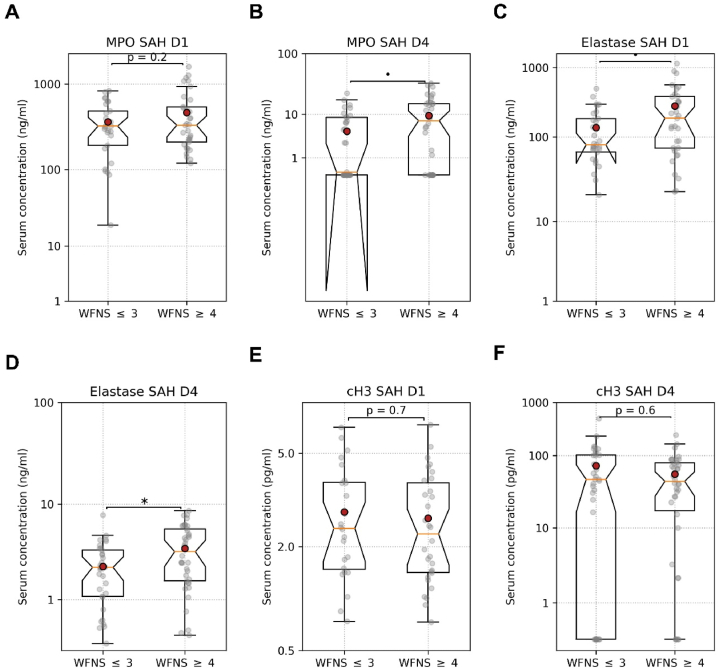
Comparison of serum levels of MPO, elastase, and cH3 between aSAH patients with WFN ≤3 and WFN ≥4 on day 1 and day 4. Serum MPO levels in aSAH patients with WFN ≤3 and WFN ≥4 (A) on day 1 and (B) on day 4. Serum elastase levels in aSAH patients with WFN ≤3 and WFN≥4 (C) on day 1 and (D) on day 4. Serum cH3 levels in aSAH patients with WFN ≤3 and WFN ≥4 (E) on day 1 and (F) on day 4. Unpaired *t*-test. •p < 0.1, ∗p < 0.05.

There was no significant difference in MPO and cH3 levels in aSAH patients with WFN ≤3 and in aSAH patients with WFN ≥4 on day 1 and day 4 after aSAH (Fig. 4A, B, E, F; Table 4). ELA levels on day 1 showed a tendency to increase, and on day 4 following aSAH had significantly higher levels in patients with poor status than in patients with WFNS ≤3 (262 ± 271 pg/ml vs. 133 ± 110 pg/ml, p = 0.03, Fig. 4C and D; Table 4). However, dichotomized WFNS levels were not associated with CVS (chi-squared test, p > 0.8).

**Fig. 5:**
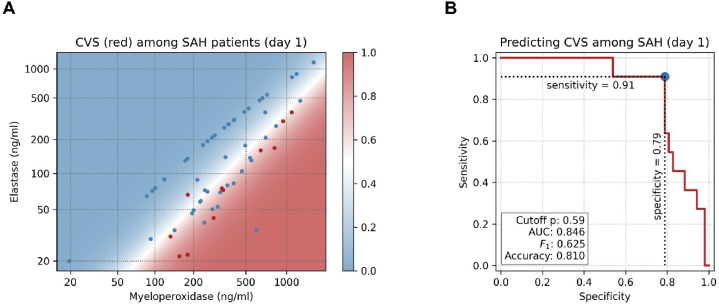
a) Measured MPO and ELA values on day one after aSAH: red = patients who developed CVS; blue = patients who were spared CVS. The colored background encodes the predicted probabilities of developing CVS (blue = low probability, red = high probability). b) Sensitivity-specificity curve for predicting CVS based on MPO and ELA values.

Taken alone, none of the ELISA values could predict CVS in aSAH patients. However, MPO and ELA together, measured on day 1, allowed for a very good prediction, with 91 % sensitivity and 73 % specificity (logistic regression, pseudo-R^2^ = 0.26, p < 0.001). Fig. 5a and b shows the separation of the classes and the corresponding sensitivity-specificity curve. By moving the separation line up, even a 100 % sensitivity could be achieved, correctly predicting all CVS cases, at the cost of losing specificity (i.e. having more false alarms). Whether this is desired remains to be decided by the clinician. The relatively large number of false positives is also reflected in the F_1_-score of 0.571 (ideally, we'd want it to be close to one). Prediction was also possible using ELA and cH3, albeit with a lower performance (pseudo-R^2^ = 0.17, p = 0.006). On the other hand, none of the ELISA values for MPO, ELA, and cH3, neither alone nor combined, could successfully predict infarctions among the SAH patients. Regarding the eventual outcome, measured on the modified Rankin Scale, only WFNS was a significant predictor (simple linear regression, R^2^ = 0.44, p < 0.001) (Table 1, Table 5). In multiple linear regression, adding ELA, MPO, and the binary indicator whether the patient suffered CVS as the predictors, WFNS was still the only significant one. Even when removing ELA and MPO, to avoid collinearities, and using only WFNS and the CVS indicator as the predictors, CVS remained highly non-significant (p = 0.99).

## 6 Discussion

It is well known that inflammation is one of the major contributors to post-aSAH complications [7]. Previous studies have shown that a high neutrophil count and high neutrophil-to-lymphocyte ratio are good predictors of complications following SAH [12,13,16]. Neutrophils can contribute to post-aSAH complications via different mechanisms, including NETosis. In this prospective exploratory study, we investigated whether components of NETs can predict cerebrovascular vasospasm after aSAH.

Interestingly, all three components of NETs, MPO, ELA, and cH3, were significantly lower in aneurysm patients than the healthy controls and patients with ruptured aneurysm (Fig. 1A–D, E, Fig. 2A–D, E, Fig. 3A–D, E; Table 3). The levels of NETs components in serum have not been investigated in intracranial aneurysm patients compared to healthy control. Previously published clinical and experimental animal studies mainly emphasized and investigated the role of NETs components in cerebral aneurysm rupture [18,19,37,38]. We could find one study reporting a higher elastase to alpha-1 anti-trypsin ratio in patients with unruptured intracranial aneurysms than in non-operative controls [39]. The higher elastase to alpha1 anti-trypsin ratio was due to increased elastase levels in patients with unruptured intracranial aneurysms than in non-operative controls [39], which is contrary to our findings. Our findings also do not agree with the observations reported by Zheng et al., showing higher cH3 levels in aSAH patients than in the control groups [29]. However, the serum levels of all three investigated components of NETs were significantly higher in aSAH patients on day 1 and day 4 than in the patients with cerebral aneurysms (Fig. 1, Fig. 2, Fig. 3; Table 3). Previously published clinical and experimental animal studies have shown higher levels of NETs components in the wall of ruptured aneurysms than in the wall of unruptured aneurysms and in the serum or plasma of the patients with ruptured aneurysms compared to the patients with unruptured aneurysms and controls, accentuating on the contribution of NETs to aneurysms rupture [18,20,38]. Korai et al. showed that blocking NETs formation or disrupting pre-formed NETs was sufficient to reduce the rate of aneurysm rupture [37]. It is remarkable to mention that elastase, one of the primary components of the NETs, is used in animal models of cerebral aneurysm formation and rupture [9]. Gounis et al. showed that half of the unruptured intracranial aneurysms were negative for MPO, while all ruptured aneurysm specimens were positive for MPO [18]. It is noteworthy that MPO-positive cerebral aneurysms have a higher risk of rupture than MPO-negative aneurysms within five years, as predicted by the PHASE model [18,40]. Chu et al. demonstrated that the formation and rupture of cerebral aneurysms were significantly reduced in MPO-deficient mice [19]. MPO and elastase can reduce the tensile strength of the walls of cerebral arteries, consequently increasing the risk of rupture. Furthermore, the components of NETs did not show a significant difference between healthy controls and aSAH patients on day 1 (Fig. 1, Fig. 2, Fig. 3B; Table 3). However, in aSAH patients on day 4, the NETs components ELA and MPO were significantly decreased than in aSAH patients on day 1 and in healthy controls (Fig. 1, Fig. 2C; Table 3). Previously, Witsch et al. reported a decrease in MPO-DNA complexes from day 1 to day 4 in aSAH patients with delayed cerebral ischemia compared to aSAH patients without delayed cerebral ischemia [41]. In our study, we also observed a significant decrease in the serum levels of MPO and ELA from day 1 to day 4 (Fig. 1, Fig. 2F; Table 3). However, we could not find a correlation between the decrease in these NETs components and cerebral vasospasm.

All three investigated NETs components, MPO, ELA, and cH3, on day 1 and day 4 after aSAH did not show a difference in patients with and without CVS (Fig. 1, Fig. 2, Fig. 3; Table 3). However, the serum levels of ELA and MPO on day 1 after aSAH taken together allowed a very good prediction of CVS in aSAH patients (Fig. 5). The predictive characteristics of ELA and MPO on day 1 have significantly high clinical relevance. The earlier prediction of CVS after aSAH can be very useful for timely intervention to take measures for preventing CVS following aSAH. Zheng et al. reported a significant correlation between plasma cH3 levels and the severity of SAH for aSAH patients [29]. In our study, serum MPO levels on day 4 showed an increasing trend (Fig. 4B), and the serum levels of ELA were significantly higher on day 1 and day 4 in patients with poor WFN scores (Fig. 4C and D). Our finding that the patient's initial status, reflected in the WFNS score, predicts the outcomes, is in line with the evidence reported in the literature [4]. Yet, it is somewhat surprising that CSV and, consequently, MPO and ELA, which predict it, have no significant effect on the outcome. Still, CSV is a dangerous complication, requiring special attention, and MPO and ELA can be utilized to predict it.

Elastase and MPO are two of the major enzymes stored in azurophilic granules of neutrophils and are the primary components of NETs. These enzymes can contribute to post-aSAH complications through different mechanisms. ELA and MPO can activate MMPs and increase the expression of MMPs, cytokines, adhesion molecules, and chemoattractants [19], through which these enzymes promote the infiltration and accumulation of inflammatory cells, resulting in enhanced inflammation. Furthermore, ELA and MPO can impair endothelial function and disrupt the blood-brain barrier [21]. The dysfunctional endothelial cells also increase the expression of pro-inflammatory markers such as TNF-α, IL-1β, and IL-6 and MMPs [[42], [43], [44], [45]]. Endothelial dysfunction is one of the earliest events followed by smooth muscle cell phenotypic switch and infiltration of innate immune cells in aneurysm formation and rupture [9]. After cerebral aneurysm induction, neutrophil infiltration, pro-inflammatory marker expression, and reduction of α-smooth muscle actin were significantly less in the cerebral arteries of MPO-deficient mice [19]. Blocking NETs attenuated the activation of microglia/macrophages, ameliorated the expression of pro-inflammatory cytokines (TNF-α, IL-1β, and IL-6), and increased the expression of anti-inflammatory cytokine IL-10 *in vivo* after SAH, aSAH, and traumatic brain injury and *in vitro* in microglial BV2 cells [16,25,29,37]. Previously published clinical data has shown the positive association of TNF-α, IL-1β, and IL-6 with post-aSAH complications and poor prognosis [7,46]. Blocking TNF-α, IL-1β, and IL-6 inhibited CVS, reduced neurological deficits, and improved outcomes after SAH in clinical and experimental animal studies [7]. In addition to NETs components, the production of ROS and release of TNF-α and IL-6 by neutrophils can also damage the blood-brain barrier [21]. The disruption of the blood-brain barrier leads to the direct exposure of brain tissue to immune cells and neurotoxic contents in blood, which subsequently causes secondary brain injuries such as oxidative stress, mitochondrial dysfunction, neuronal degeneration, and exacerbation of inflammation [23]. In addition to that, MPO and ELA can induce oxidative stress [27], which can also result in neuronal degeneration, endothelial dysfunction, and loss of SMCs [47,48]. Also, MPO can promote endothelial dysfunction and vasoconstriction by consuming nitric oxide (NO) as a substrate, reducing eNOS activity, and decreasing eNOS Ser1177 phosphorylation [27,49,50]. Previous studies have demonstrated that blocking NETs formation improved neurological impairment, inhibited microthrombosis, brain edema, neuronal injury, and blood-brain barrier disruption, and reduced the incidence of DCI [16,22,25,26,[29], [30], [31]].

Our current data, in light of already published clinical and experimental animal studies, suggest that myeloperoxidase and elastase are significantly important markers for cerebral vasospasms with high clinical relevance.

## 7 Conclusion

Elastase and myeloperoxidase are two easy-to-determine markers that are highly predictive of cerebral vasospasms in patients suffering from aSAH. They can be used as early warning signs and to adjust therapy and patient management.

## 8 Limitations

It can be argued that linear regression is not a suitable tool to model an ordinal outcome variable, like mRS, and that an ordinal model should have been used. This critique would certainly have its merit if the purpose of this study were to predict the outcome. However, as our continuous-valued variables showed not to be significant predictors, it sufficed here to show that the patient's initial status, as reflected in the WFNS score – again an ordinally-scaled variable – is strongly associated with the outcome, without delving into the details of the relationship. Moreover, the NETs measurements were performed in serum collected within 24 h and 4 days after the aSAH onset, which might not capture the dynamic changes of NETs during the disease course after aSAH. The NETs measurements at later time points after aSAH would better track the trajectory of these biomarkers and would provide detailed information on the role of NETs in the progression of CVS.

## CRediT authorship contribution statement

**Saba Sajjad:** Writing – original draft, Methodology, Investigation, Formal analysis. **Michael Hewera:** Investigation, Data curation. **Majeed Rana:** Writing – review & editing. **Michael Gliem:** Writing – review & editing. **Igor Fischer:** Visualization, Investigation, Formal analysis, Data curation. **Dilaware Khan:** Writing – review & editing, Supervision, Methodology, Conceptualization.

## Availability of data and materials

Data is provided within the manuscript or supplementary information files.

## Ethical approval

All procedures performed in the study involving human participants were in accordance with the ethical standards of the institutional committee and with the 1964 Helsinki Declaration and its later amendments. The study was approved by the ethics committee of the Medical Faculty of Heinrich-Heine-University, Germany ((Studien-Nr.: 2019-787-bio)). Written consent was needed for participation in this study. The written consent of patients capable of giving informed consent was obtained immediately. Incapacitated patients were initially enrolled in accordance with the ethics approval, and consent was obtained from the patient themselves or their authorized representative at the earliest possible time.

## Consent for publication

Not applicable.

## Funding

The internal funding of the Department of Neurosurgery, University Hospital Duesseldorf was used.

## Declaration of competing interest

The authors declare that they have no known competing financial interests or personal relationships that could have appeared to influence the work reported in this paper.
